# Effects of Fatigue Based on Electroencephalography Signal during Laparoscopic Surgical Simulation

**DOI:** 10.1155/2018/2389158

**Published:** 2018-05-02

**Authors:** Nyakuru Z. Ndaro, Shu-Yi Wang

**Affiliations:** Department of Biomedical Engineering, School of Medical Instrument and Food Engineering, University of Shanghai for Science and Technology, Shanghai 200093, China

## Abstract

**Background:**

Following recent advances in technology, there is a growing interest in studying fatigue based on electrophysiological signals as a means of monitoring brain activity. While some existing works relate fatigue to performance, others consider the two as independent entities. Therefore, we must explore this intricate issue, particularly in laparoscopic training, for the sake of patient safety.

**Objective:**

This paper explores and evaluates effects of fatigue on efficiency and accuracy based on laparoscopic surgical training using Electroencephalography (EEG) signal.

**Materials and Methods:**

20 college students performed peg transfer task on laparoscopic simulator, with real-time recording of EEG signals for each subject. To monitor degree of fatigue, a real-time fatigue monitoring system based on fatigue analysis algorithm was designed through the use of EEG in alpha (*α*) and theta (*θ*) rhythms. We designed data acquisition and fatigue analysis modules based on MATLAB platform. BrainLink was used to record EEG signals and send them to personal computer wirelessly via Bluetooth. While artifacts from the captured EEG signals were removed using Blind Source Separation (BSS), *α* and *θ* rhythms were extracted using wavelet analysis. Fatigue was evaluated based on Regression Model and Mahalanobis Distance (*D*_*C*_), and its threshold was determined from the experimental results using Receiver Operating Characteristic (ROC) curve analysis.

**Results:**

Completion time and number of errors behaved like a decreasing function during the first few trials while increasing afterwards with the increasing of perceived fatigue level. The results indicate that learning curve of the subjects is increasing until 13th trials when they have attained maximum learning benefits and decreases afterwards due to fatigue.

**Conclusion:**

Regression analysis shows that there are significant learning and fatigue effects when peg transfer task in the training is repeated in a series of trials. However, for the training to be effective and efficient, there should be monitoring during the training to observe where in the learning curve a trainee gains maximum learning benefits. Furthermore, fatigue is a significant indicator of efficiency and accuracy in terms of completion time and errors, respectively.

## 1. Introduction

Surgeons, particularly those in training, work for a long period of time and are often sleep deprived [1]. Such personnel may possibly experience disturbance in circadian rhythms, which may exacerbate physical or mental fatigue. Fatigue has been proved to decrease efficiency in cognitive skills [2], leading to decrease in performance as well as increasing rate of medical errors [3, 4]. However, previous studies that investigated the link between performance and fatigue are conflicting. Some studies concluded that fatigue has no impact on surgical performance [5, 6] while others link it to performance [7, 8]. Therefore, understanding the effects of fatigue, particularly in surgical training, remains an open problem.

In surgical field, virtual reality surgical simulation has been an alternative approach to examine effects of fatigue on performance [7–9] without reliance on patients for laparoscopic training. The surgical simulator is a cost-effective laparoscopic training device that uses innovative learning tools for skill acquisition and can differentiate user's level of experience. It can also evaluate surgical performance without the use of patients for skills practice [9]. A number of studies used surgical simulators to investigate effects of fatigue in laparoscopy. For example, Daruwalla et al. investigated effects of fatigue on laparoscopic skills and found that fatigue has effects on laparoscopic tasks such as peg transfer [7]. This finding was supported by Tsafrir et al. [8] who argued that efficiency and safety of residents are affected by fatigue [8]. Yet, most of the existing works are based on indirect way of measuring brain activities. These include the use of individual report scaling questionnaires [4], eye blink pattern using camera systems or Electrooculography (EOG) [10], and heart rate variability by Electrocardiography (ECG) [11]. However, an individual report questionnaire lacks sensitivity in detection of small important changes in mental fatigue. In EOG, the closure of eyelid increases with increasing fatigue while ECG holds a reflection of autonomic nerve activity. Conversely, it is very important to monitor brain activity using direct measurement technique such as Electroencephalography (EEG) [11]. This technique is regarded as a promising method for detecting mental fatigue and provides the most direct measurement of brain activity [11]. Therefore, this paper explores and evaluates effects of fatigue on efficiency and accuracy based on laparoscopic surgical simulation using Electroencephalography (EEG) as a means of monitoring brain activity. In order to study this precisely, a real-time fatigue monitoring system based on fatigue analysis algorithm was designed and 20 college students were employed to perform laparoscopic task, with real-time recording of EEG signals for each subject.

## 2. Materials and Methods

### 2.1. Subjects

20 healthy college students in the age range of 24 to 26 with correct visual acuity of 1.0 or more were recruited from University of Shanghai for Science and Technology. A lifestyle questionnaire was administered and used as a selection criterion which required the subjects to have no medical contraindications such as history of prior brain injuries, use of prescription medication, severe concomitant disease, drug abuse, or alcoholism as well as psychological or intellectual problems, which more likely limit compliance.

All subjects filled informed agreement form and none of them had previous experience with laparoscopic simulator. Likewise, all subjects received comprehensive instructions about the task described in Section 2.3 and were given 15 minutes of hands-on introduction for orientation with the equipment until they became competent in laparoscopy simulation task. This study had the approval from the institutional ethics committee. Subjects were requested to stop cigarette smoking and consuming caffeinated drinks or any other stimulant 48 hours prior to the experiment as they may interfere with the trend of fatigue, and compliance with these instructions was reported. The experiment was performed from 13:00 to 17:00 hours in a temperature controlled room with continuous peg transfer task 20 times, in order to make the subjects into fatigue state.

### 2.2. Real-Time Fatigue Monitoring System

We designed a real-time monitoring system to evaluate and analyze effects of fatigue on efficiency and accuracy during laparoscopic simulation. The system consists of EEG data acquisition module, fatigue analysis module, Bluetooth communication module, and laparoscopic simulator platform. The data acquisition and fatigue analysis modules were designed based on MATLAB platform.

#### 2.2.1. EEG Signal Recording and Acquisition Module

**Figure 1:**
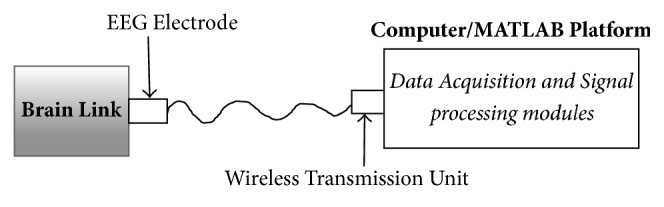
Schematic view of EEG fatigue monitoring system.

The experiment employed a virtual reality laparoscopic simulator (called Simbionix LAP Mentor) to perform peg transfer task. This simulator has a wide variety of modules ranging from simple to complex laparoscopic tasks such as suturing, basic operation, cholecystectomy, gastric bypass surgery, incision hernia surgery, gynecological surgery, rectal surgery, and laparoscopic assembly skills. During the experiment, BrainLink was used to record EEG signals through the use of its three forehead electrodes. The recordings of neuronal activity in the brain are identified as EEG signals [12]. Indeed, the electrodes read EEG signals from head surface and send them to personal computer wirelessly via Bluetooth module for storage and display, as demonstrated in Figure 1.

#### 2.2.2. Signal Processing and Fatigue Analysis

**Figure 2:**
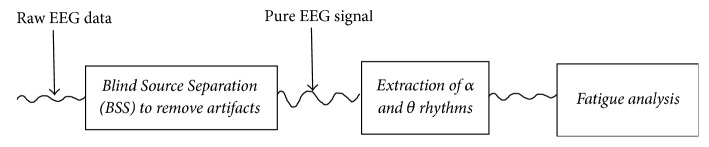
Three steps for signal processing and fatigue analysis.

Signal processing and fatigue analysis were designed to follow three steps, as demonstrated in Figure 2.

**Figure 3:**
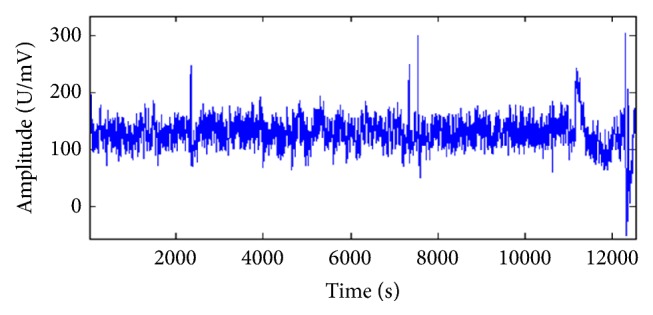
Raw EEG signal from the BrainLink.

The first step is to remove artifacts from EEG signals. Although BrainLink is designed to record cerebral activity, it also records electrical activities arising from entities other than the brain. Generally, any activity that is recorded apart from cerebral origin is termed as artifact. In most cases, the artifacts can be obtained from either physiologic or extra physiologic perspectives. The former are unwanted physiological signals that arise from source other than the brain, that is, the body. For example, eye movements, heart, and muscles. The latter are technical and arise from outside the body, for example, noise in AC power line which can be reduced by decreasing electrode impedance and shorter electrode wires. In fact, any EEG signal greater than 50 *μ*V is regarded as an artifact [13, 14]. However, many artifacts cannot be identified if their amplitude is lower than 50 *μ*V. Therefore, a systematic approach to eliminate artifact is important to reduce chance of misinterpretation and analysis of the EEG signal. Several techniques have been proposed to remove artifacts from EEG recording. In the present study, a Blind Source Separation (BSS) method is employed to remove artifacts from EEG data. BSS is a signal processing method that includes independent component analysis. BSS technology separates interfering signal from the original. By studying distribution of the artifacts and the use of BBS technology, we obtained a pure EEG signal from the raw EEG data. The raw EEG signal obtained from the BrainLink is demonstrated in Figure 3.

**Figure 4:**
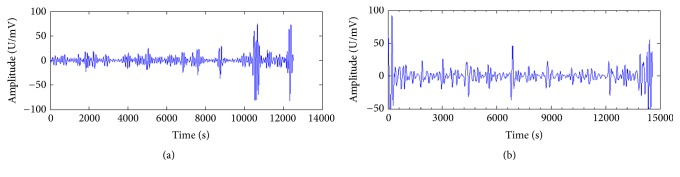
Brain waves belonging to alpha (*α*) and theta (*θ*).

The second step is the extraction of characteristic rhythm waves. Human brain pattern poses regular oscillations which are termed as rhythms and are differentiated on the basis of signal's frequency [15]. Rhythms of electrical activity in the brain are categorized according to frequency bands, namely, alpha (*α*), beta (*β*), theta (*θ*), and delta (*δ*) [4]. These rhythms are usually identified by frequency and amplitudes. The amplitudes recorded by scalp electrodes from the BrainLink are in the range of microvolts (*μ*V). The present experiment employed wavelet analysis to extract two characteristic rhythms waves: alpha (*α*) and theta (*θ*), as shown in Figure 4. Since variations in the alertness state induce changes in EEG spectra, the spectra in *θ* rhythm (4–7 Hz) and *α* rhythm (8–11 Hz) will reveal changes in cognitive state and memory performance so that when mental fatigue increases, the relative power of *α* and *θ* rhythms decreases [16].

The third step is computation of deviation of EEG signals in *θ* and *α* rhythm. When fatigue occurs, both *θ* and *α* rhythms change significantly [10] and the subject's EEG spectra in them deviate from his or her alert state. Hence, his or her cognitive state and memory performance will change. This deviation was calculated using Mahalanobis Distance (*D*). The EEG spectra in *θ* and *α* rhythms are characterized by (*μ*, *N*), where *μ* is the vector of mean values and *C* is the covariance matrix of EEG spectra. The Mahalanobis Distance is calculated as follows:

(1) Dxα=xα−μTC−1xα−μ1/2,Dxθ=xθ−μTN−1xθ−μ1/2,

where *x*_*θ*_ and *x*_*α*_ are EEG signal in *θ* and *α* rhythms, respectively. Then, the combined deviation of EEG is given by

(2) $$\begin{array}{c} D_{C}=\sigma *D\left(\;x_{\alpha }\;\right)+\left(\;1-\sigma \;\right)*D\left(\;x_{\theta }\;\right),\;0\leq \sigma \leq 1. \end{array}$$

The parameter *σ* is a constant. Since Mahalanobis Distance in *θ* and *α* rhythms changes with fatigue state, the weights of *D*(*x*_*α*_), *D*(*x*_*θ*_), and *D*_*C*_ are used as indicators of fatigue. In the present work, the threshold of fatigue was calculated from the experimental results based on Receiver Operating Characteristic (ROC) curve analysis. When the value of *D*_*C*_ is larger than the defined threshold, the cognitive state of the subject can be regarded as fatigue state.

#### 2.2.3. Software Platform

**Figure 5:**
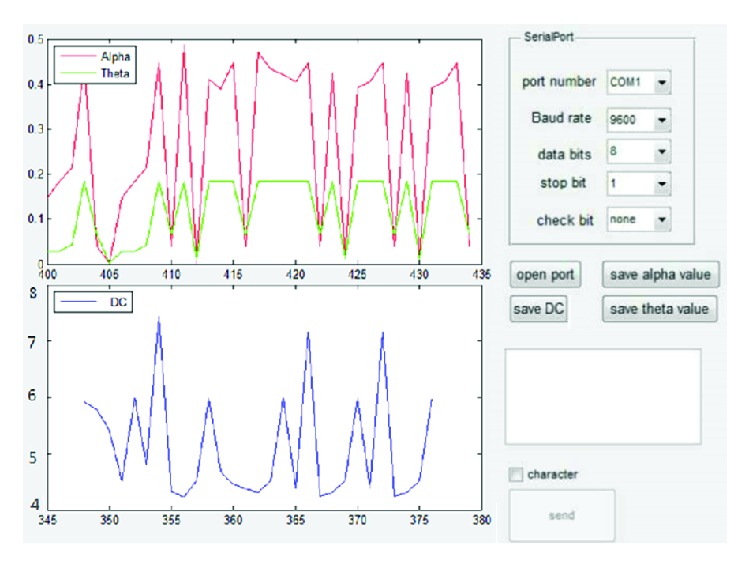
GUI platform for signal processing.

The system was designed to use GUI platform which enables functions such as data processing and drawings. The system collects EEG signals and extracts characteristic rhythms wave *α* and *θ*, in order to calculate fatigue value. Since the distance between EEG and the rhythms wave increases gradually with the increase of fatigue, it is very important to determine an appropriate threshold and compare the threshold with the weighted Mahalanobis Distance of *θ* and *α* rhythm. Based on the ROC curve analysis method, the fatigue threshold was determined (6.0 *μ*V). In the present work, when fatigue value exceeds the predefined threshold value of 6.0 *μ*V, fatigue is detected and the system immediately rings the alarm. The system interface is shown in Figure 5.

### 2.3. Experimental Procedures

**Figure 6:**
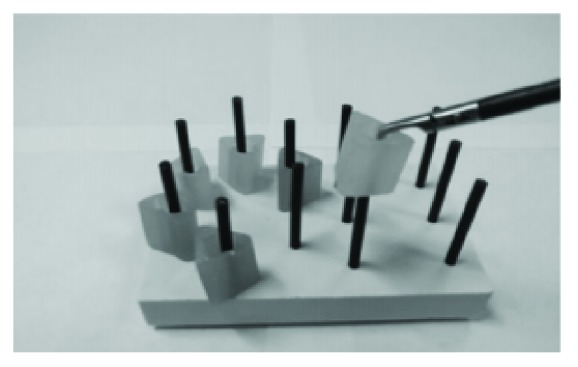
Left and right shift of six rubber triangles.

Peg transfer task requires pegboard (with 12 pegs) and six rubber triangles (Figure 6). Each triangle is lifted using a grasper in the subject's left hand and transferred in mid-air to a grasper in his/her right hand. Then, the triangle is placed on a peg on the opposite side of the board. After moving all the triangles on the right side, then every triangle has to be moved back to the left side of the board, using the same procedure. The number of errors is defined when a subject drops the triangle out of reach. The maximum completion time was set to 300 seconds in the simulator.

## 3. Results

The training was evaluated objectively by comparing efficiency (in terms of completion time), accuracy (in terms of number of errors), and fatigue level.

### 3.1. Objective Indicators of Effective Laparoscopic Surgical Training

In most laparoscopic surgical training, minimizing number of errors, completion time, and fatigue level is preferred in general as it would improve efficiency of the training. To get representative sample of each aforementioned quantities, we averaged the results of all subjects at each training trial. Herein, we present the results of errors made during peg transfer task, time to complete the task, and fatigue level.

#### 3.1.1. Number of Errors

**Figure 7:**
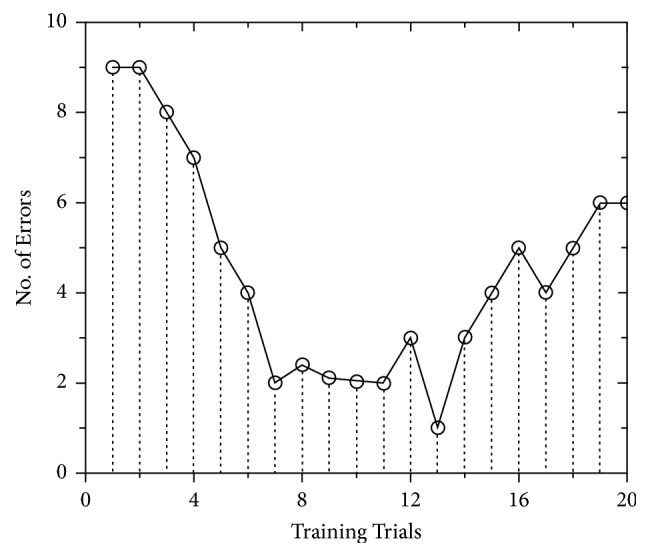
Number of errors during peg transfer task.

Regression Model predicted that the number of errors made during the first seven training trials was associated with lack of technical skills and perhaps new procedures [17], while it was associated with fatigue at the last eight trials. As predicted by the linear regression equation *y* = 12.13 − 1.4 × (*R*^2^ = 0.98), the number of errors decreases until the seventh trial where it quickly attains a stable degree upon which the competency of the subject is anchored, as observed in Figure 7. This indicates that learning curve of the subjects has increased until seven trials when they have attained maximum learning benefits. During the 8th to 11th trials, the average number of errors is two, which is lower than the first seven, meaning that the subjects have acquired enough skills to cause fewer or no errors. The linear fitting equation portray *y* = 3.34 − 0.13 × (*R*^2^ = 0.7), indicating a decreasing trend; however, such decrease is very scant. Between 13th and 20th trials, the number of errors is increasing and the function runs uphill, when a subject is acutely fatigued, with linear regression equation of *y* = −5.96 + 0.62 × (*R*^2^ = 0.796). This means that maximum learning benefits will be gained by subjects until 13th trials and decrease afterwards due to fatigue. From the regression equation, it is shown that the slope of the regression line is 0.62 and the number of errors is expected to increase by 0.62 on average when the training proceeds to the next trial. Being fatigued significantly increases the chance of errors and decreases the learning curve. Therefore, when the same task in the training is repeated in a series of trials, there should be monitoring to observe where in the learning curve a trainee gains maximum learning benefits and then stop the training.

#### 3.1.2. Completion Time

**Figure 8:**
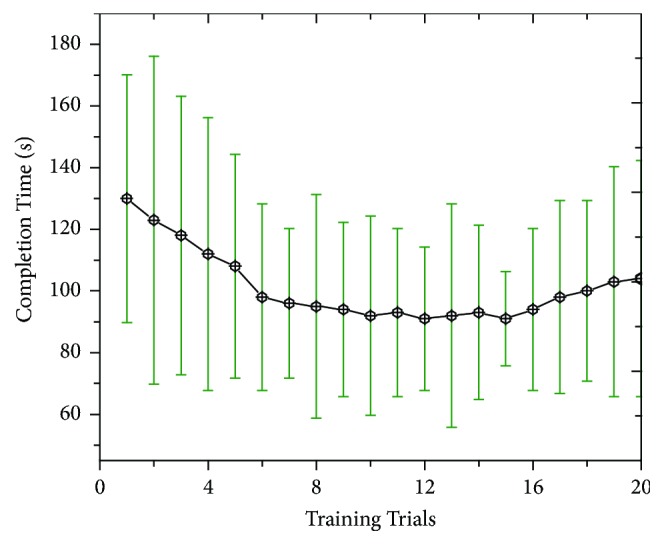
Completion time as a function of training trials.

In terms of completion time, the laparoscopic simulator also calculated time spent for each subject to complete peg transfer task. The results show that all subjects took long time to finish the peg transfer task at the first time (around 130 seconds). However, each subsequent time they did the task took less time than the previous one until around 12 trials. This indicates that the subjects have learnt from doing peg transfer task and became faster each time they repeated the task. Nevertheless, the completion time started to increase at later trials due to fatigue effect. Precisely, during the first few training trials, the subjects took long time to complete the task. This is when they were acclimatized with the training. As more and more trials were conducted, significant improvement was found and the completion time decreased by 24% as predicted by the regression equation *y* = 2.1 − 1.4 × (*R*^2^ = 0.87). However, the completion time remained stable at later trials until the 14th trial, which is shorter than the other trials. This is when the subjects have acquired enough skills to perform more effectively. When fatigue was drawn after 14 trials, the completion time was increased from 92 to 110 seconds, as shown in Figure 8. These results have indicated that there exists significant learning for repeated peg transfer tasks.

#### 3.1.3. Rate of Fatigue

**Figure 9:**
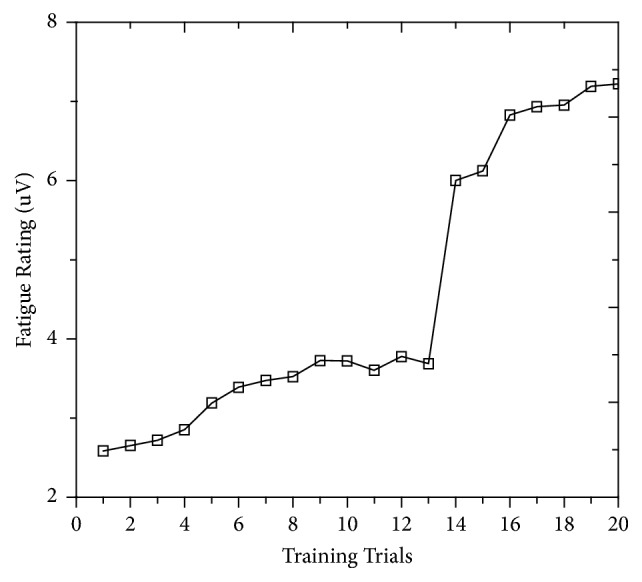
The measured rates of fatigue.

Fatigue exhibited different behavior, with its rate growing gradually up to around ninth training trials, as predicted by the linear regression equation *y* = 2.35 + 0.15 × (*R*^2^ = 0.96), and then the trend started to fluctuate around 3.6 until 14th training where it increased sharply, as shown in Figure 9. This means that subjects are fully alerted, their self-regulatory capacities are normal, and they can concentrate on the training until 13th trials. Thereafter, the subjects undergo ego depletion and their capacities become exhausted [18]. It can be seen that the rate of fatigue was increased by 62.8% from 13th to 14th trials, which indicate that excessive number of training times reduces efficiency. During 14th to 20th trials, fatigue values behaved like an increasing function and run uphill to seven, with regression equation *y* = 3.15 + 0.212 × (*R*^2^ = 0.84).

## 4. Discussion

Prolonged working hours and lack of sleep have been associated with loss of attention, performance decrements, and increase in errors in medical practitioners [19–21]. Following these findings, some countries had set some regulations regarding the working hours for physicians. For example, in Scandinavia the shift work is restricted to 16–24 hours and the physicians have the day off after a night shift. In United Kingdom, it is allowed to work for a maximum of 16 hours in 24 hours [22]. In United States, interns are limited to a maximum of 16 hours per shift in the 80 hours' work week [6]. The residents association in Canada (Fédération des médecins résidents du Québec) had set a limit of maximum 16 hours of consecutive working due to the fact that 24-hour continuous work endangers residents' health [23]. In the present study, regression analysis indicates that working for a long period of time induces fatigue that can increase number of errors and time to complete peg transfer task during laparoscopic simulation. These results are consistent with what was found in the previous studies [6, 9]. However, they are very different from those of Bagrodia et al. [24] and Uchal et al. [5], who detailed that fatigue and performance are covariates. Increase in errors and time to complete laparoscopic task is more likely to be caused by trainee during acclimatization; however, as more and more trials are performed, the learning curve increases, which gradually decreases errors and completion time. Yet, when fatigue is developed, the number of errors and completion time start to increase again. Nevertheless, the consequence is more severe during the acclimatization than when being fatigued after making many trials. Though, sometimes, it does not matter how long it takes the trainee to complete the task; if he/she can operate without errors, then such surgical procedure can be considered as safe. It is important to understand that subject is novice during the first few trials and learns gradually as more and more trials are performed. In fact, poor performance during the first few trials was probably a reflection of new procedures [17] and of fatigue afterwards. The results support the regulation of work hour's restrictions for surgical residents, and further they emphasize that medical educators should address these negative effects of fatigue in medical students.

## 5. Conclusion

This paper has explored and evaluated effects of fatigue on efficiency and accuracy during laparoscopic surgical training using direct measurement of brain activity. The findings indicate that there are significant learning and fatigue effects when peg transfer task in the training is repeated in a series of trials. However, for the training to be effective and efficient, there should be monitoring to observe where in the learning curve a trainee gains maximum learning benefits. Moreover, fatigue is a significant indicator of efficiency in terms of time to complete laparoscopic task and accuracy in terms of errors made while doing the task. Even though these results reflect laparoscopic surgical training, the principles also apply to surgeons during patient operation, which provides some useful fundamental lessons for workplace or in hospital. Future work should entail investigating effects of fatigue in surgeons during laparoscopic surgery simulation based on Electroencephalography.
